# Understanding China’s urban system evolution from web search index data

**DOI:** 10.1140/epjds/s13688-022-00332-y

**Published:** 2022-03-28

**Authors:** Hao Guo, Weiyu Zhang, Haode Du, Chaogui Kang, Yu Liu

**Affiliations:** 1 Institute of Remote Sensing and Geographical Information Systems, School of Earth and Space Sciences, Peking University, Beijing, China; 2 Beijing Key Lab of Spatial Information Integration and Its Applications, Peking University, Beijing, China; 3 National Engineering Research Center of Geographic Information System, China University of Geosciences (Wuhan), Wuhan, China; 4 State Key Laboratory of Media Convergence Production Technology and Systems, Beijing, China

**Keywords:** City attractiveness, Spatial inequilibrium, Web search, Gravity model, Particle swarm optimization, China

## Abstract

The spatial inequilibrium phenomenon is apparent during China’s rapid urbanization in the past four decades. As the fertility rate decreases and the population ages, this phenomenon will become more critical. To accurately forecast the future economic development of China, it is necessary to quantify the attractiveness of individual cities. This study introduces web search data to quantify the attractiveness of cities with a fine spatial scale (prefecture-level city) and relatively long-term span (nine years). Results confirm that the estimated city attractiveness can unravel a city’s capability to attract labor force, and suggest that tourism and health care functions of a city have a positive effect to the city’s attractiveness. Additionally, China’s north-south gap in economic development has been widened in the past decade, and 11 cities with nationwide influence have prosperous development potential. This study provides a new lens for predicting China’s economic development, as well as its spatial patterns.

**Supplementary Information:**

The online version contains supplementary material available at 10.1140/epjds/s13688-022-00332-y.

## Introduction

China has experienced a rapid economic growth and urbanization in the last 40 years [1], during which population growth has been a major driver and will continually contribute to the future change of the nation’s urban systems. However, the recent shrinking of labor force (due to the low fertility rate) in China implied a blurry vision of the future, making it a necessity to foresee its economic development from a geographical perspective. While seeing a dramatic increase of the urbanization rate from 19.39% in 1980 to 60.60% in 2019 [2], China’s regional inequality in urbanization also becomes apparent. Many giant cities like Shenzhen have emerged, meanwhile, lots of cities such as Harbin and Shenyang are declining. It is crucial to predict the prospects of individual cities through estimating migrations between cities, which is a manifestation of a city’s capability to attract labor forces, especially young workers to sustain its future development [3].

At the national scale, the geography of Chinese cities’ attractiveness has been constantly changing, although the pace was not too fast. Such change is reasonable and expectable because the attractiveness of a city is a synthetic indicator of many factors including wage, house price, culture, and climate conditions. Even a subtle factor such as local cuisine or air quality might lead a person to move to or flee from a city. Examples include that many of the young generation have been fleeing from Beijing and Shanghai, the two most attractive cities in China about ten years ago, due to unaffordable living expenditures [4]. Consequently, the hinterlands of many central cities have also seen marked changes, unfolding another facet of the dynamics of individual cities’ attractiveness.

To understand the attractiveness of cities, traditional approach mainly relies on survey data such as socio-economic statistics indicators [5], which often suffer from incompleteness, unreliability and inconsistency across cities. Being assisted by the ubiquitous awareness of interactions between cities, a network-based perspective has been increasingly adopted to precisely estimate the attractiveness of a city. As the Internet gradually becomes an essential part of our daily life, web search traffic has been taken as a promising data set for urban studies, especially for tourism prediction [6–9]. Inter-city web search volumes, which quantify the frequency that a city’s name being searched by users of another city for the sake of either business, employment, tourism, or simply curiosity, can be taken as the proxy data of spatial interaction intensity between cities, so as to provide us a new lens to investigate the attractiveness of cities.

Based on longitudinal web search volumes, this study estimates the attractiveness of individual cities in China over the last decade by reversely fitting the gravity model, and investigates its spatio-temporal patterns. The estimated city attractiveness is yielded from a novel directed gravity model with particle swarm optimization, and can provide us with reasonable evidences to foresee the future development of the nation’s urban systems.

## Related works

Measuring the attractiveness of cities has been a central topic in geography and regional studies for a long time [10]. Previous studies have suggested that the determinants of city attractiveness can be mainly summarized as six functional dimensions: economy, research and development, cultural interaction, livability, environmental and accessibility [11]. Along with the general studies of city attractiveness, tourist cities and touristic attractiveness have also been extensively studied [12–14]. In earlier studies, researchers often take population size, economic scale, or city area as the quantitative descriptor of city attractiveness [15, 16]. This simplification was supported by empirical evidence showing that population sizes, used per capita income, percentage of tertiary sector, tourism revenue per capita and railway dominance well explain the discrepancies between the developments of cities [17]. However, the relationship between those economic/demographic indicators and city attractiveness might be very complex to decouple. For instance, researchers have found a super-linear scaling law of city attractiveness with population size for foreign tourists [18]. Whereas for residents, with the improvement of social economic level and increasingly prominent environmental problem, cities with large populations might not be the most attractive places to live in. As big cities are often accompanied by higher living costs and inevitable “urban disease”, some second-tier cities might tend to be more attractive for young populations. Hence, attractiveness of current cities is not driven by jobs or economics only, factors concerning the direction of population movement are playing increasingly important roles in defining the attractiveness of a city [19–21].

Alternatively, reversely fitting the gravity model on city interactions becomes a commonly used approach to reconstruct the attractiveness of cities. The gravity model (also known as the spatial interaction model) is an important method for urban and regional analysis. There is a rich body of literature on the model and its various extensions, termed the family of gravity models [22, 23]. In its generic form, the use of gravity model is to predict the interaction intensity given the city sizes and the distances. Yet it also provides a tool to estimate the theoretical sizes of a set of cities, known as gravitational attractions, under the scenario that the interaction flows and distances between each city pair are known. This reversely fitting approach (referred to as reverse gravity model) has been widely used for estimate city attractiveness in various types of spatial interactions, including air passenger flows [17, 24, 25], social media check-ins [26, 27], toponym co-occurrences [28] and tourism flows [14]. For example, Xiao et al. [17] reconstructed the gravitational attractiveness of 25 major cities in China in 2001 and 2008 from air passenger flow. Liu et al. [28] estimated theoretical sizes of Chinese provinces based on toponym co-occurrences. He et al. [27] inferred the attractiveness of 348 prefecture-level Chinese cities from the social media check-in data.

The existing methods for reversely fitting gravity models can be divided into two categories, exact methods and heuristic methods. Based on the mathematical expression of the reverse gravity model, exact methods includes three existing approaches: linear regression, linear programming (LP), and the algebraic method. O’Kelly et al. [24] applied linear regression and LP to derive the gravitational attractions of 25 major cities in the United States from the air passenger flow data in 1970. Using the same data set, Shen [25, 29] developed the algebraic method and its simplified version that can solve the fitting problem with lower computation complexity. Although each of these exact methods has its merits, all have some limitations in reaching a fast and accurate solution on large network data [30]. LP faces challenges on both computation time and stability when applied to a large spatial interaction network. The algebraic method is defective as edges with zero flow need special treatment. Therefore, their applications have been scarce in recent studies. Heuristic methods such as the particle swarm optimization (PSO) method offer better ability in solving the reverse gravity model. Xiao et al. [17] first utilized PSO to solve reverse gravity model, and compared the performance of different approaches on air passenger flow networks. Results show that PSO outperforms LP and algebraic method in all cases with different network size and sparseness. Moreover, PSO does not require the objective function in any particular form as in LP, which needs a logarithmic transformation of the gravity model; nor assumes null flow between cities as a constant as algebraic method does. Therefore, it has more flexibility in solving reverse gravity models.

There is much to learn from understanding the discrepancies between the estimated gravitational attractions (“theoretical”) and actual city sizes. City attractiveness reported in previous studies heavily relies on a single type of spatial interactions in the physical space [14, 17, 24–27]. Taking transport network as an example, it is easy to overestimate a city’s attractiveness by one type of transport mode if the reliance of a city on that mode is high. In contrast, we may underestimate a city’s attractiveness under situations that other transport systems have a strong influences on the city’s interactions with others. Such discrepancies suggest that the estimated city attractiveness from one mode need to be interpreted in a multimodal context to understand the complementary roles of various transportation modes in urban development. Other factors such as income, economic structure, and geography might also influence travelers’ mode choices and consequently the market share of a transport system. As the World Wide Web has become a vast knowledge base, such Internet-based data provide a fresh perspective on the real world when compared with traditional geospatial data. A few studies have already developed new approaches to investigate the relation between geographical entities from data collected from massive web pages, such as toponym co-occurence [28]. Compared with common interaction data such as inter-city flight volumes, web data demonstrate its superiority in the following aspects: (i) Wide spatial coverage. The data have nationwide coverage and are not constrained by traffic networks. In comparison, traffic flow data such as air passenger flow data can only be used to analyze major cities with airports; (ii) High temporal resolution and long-time span. The data is updated daily and accumulated across years, enabling us to analyze the variation and evolution of city attractiveness. It is noteworthy that, as an emerging data source, the advantages and disadvantages of web documents are widely debated concerning internet penetration rate, toponym ambiguity, and temporal variation.

## Data

Baidu Index [31] released by Baidu Inc. is our main data source for estimating city attractiveness. As the largest Chinese search engine around the world with widespread users in mainland China, Baidu publishes its web search index that counts the times a keyword being searched by Baidu users in certain area each day, and is updated daily since 2011. We may obtain the search index from one city to another by setting the keyword and user area. To query the index from Beijing to Shanghai, for example, set the user area to Beijing and the keyword as “Shanghai” (in Chinese). In this case, we call Beijing the source city and Shanghai the target city. We opt for web search index to derive city attractiveness due to not only its wide spatial coverage and high temporal resolution, but also its rich semantics, that is, people attracted by a city may not essentially have an actual visit to that city, while such attraction may be reflected by their web searching activity, so as in the web search index data.

**Figure 1:**
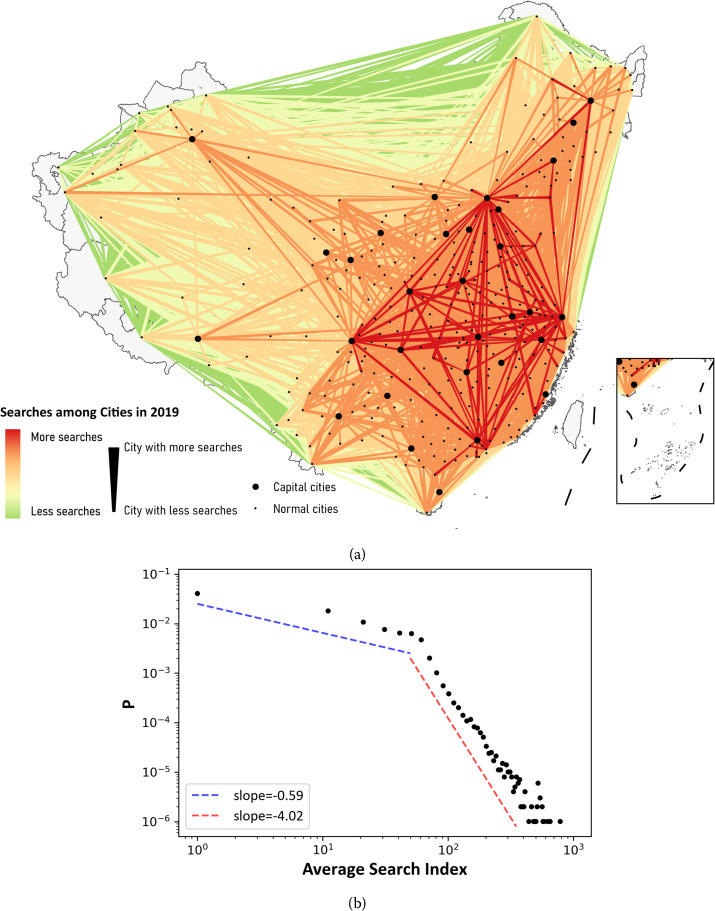
Characteristics of searches between cities in 2019. (a) The map of searches between city pairs in 2019. (b) Probability distribution of edge weights between cities in 2019

The aim is to estimate the attractiveness of cities in China for each year from 2011 to 2019, and the seasonal variation is not our focus. For each city pair, the daily search index data from 2011 to 2019 are collected, and averaged annually to avoid the influence of seasonal variation. To leave out the influence of COVID-19 on web search since 2020, we concentrated on city attractiveness before 2020 for the sake of reliability. To cover mainland China (i.e. excluding Hong Kong, Macao and Taiwan) as much as possible, we try to include all prefecture-level cities as well as county-level cities directly under provincial government, except for those whose names have not been included as keywords or not available as user areas. As a result, our city list covers 333 prefecture-level cities in mainland China, excluding Sansha in Hainan province due to lack of data. We also cover all four municipalities directly under the central government, namely Beijing, Tianjin, Shanghai and Chongqing as well as 20 county-level cities in our study. A total of 357 cities are considered from 2017 to 2019, while from 2011 to 2016, only 322 cities are covered due to data incompleteness. From the Baidu Index data between city pairs, we construct a directed network to depict the searching behaviors among Chinese cities for each year. An example of 2019 is presented in Fig. 1a. The edge weights of the network follow a broken power law distribution (Fig. 1b) and basic descriptive statistics for the edge weights is shown in Supplementary Table 1 in Additional file 1.

In addition, we evaluate two general issues concerning the web search activity. The first is toponym ambiguity [28]. Cities with ambiguous names sometimes have abnormally high search index values (see Supplementary Discussion 1 in Additional file 1 for a detailed discussion). Due to the difficulty to detect and eliminate ambiguity systematically, we did not modify our data for this issue, but considered its influence when interpreting our results. The second is the impact of public emergencies. We found that a city’s search index may increase to up to a hundred times of the normal value after the occurrence of a public emergency that draws nationwide attention. A large proportion of these events are negative, like earthquakes, explosion hazards and so on. We suppose that high values caused by public emergencies cannot be regarded as evidence for an attractive city, and values on normal days reflect city attractiveness better. Therefore, we ignored search index values higher than 4 times the year average. This threshold is based on our observation of the data that normal fluctuation of search index (caused by tourism peak season and off season, for example) seldom exceeds 4 times the year average. Thus it is reasonable to treat these values as abnormal.

It is also worth noting that, according to China Internet Network Information Center (CNNIC), the Internet penetration rate in China is 64.5% in total and 76.5% in urban areas in 2019 [32]. In 2018, most provinces in China had an Internet penetration rate of over 50% (see Supplementary Table 2 in Additional file 1), and the Internet penetration rate in China is still in the process of rapid increasing. Therefore, search index data could reflect the interests of most urban residents, and it is possible to derive reliable city attractiveness from it. For those without network access, it is natural that their concern for other cities and willingness to migrate are weak as well. This mitigates the impacts of sample bias on estimated attractiveness.

## Methods

**Figure 2:**
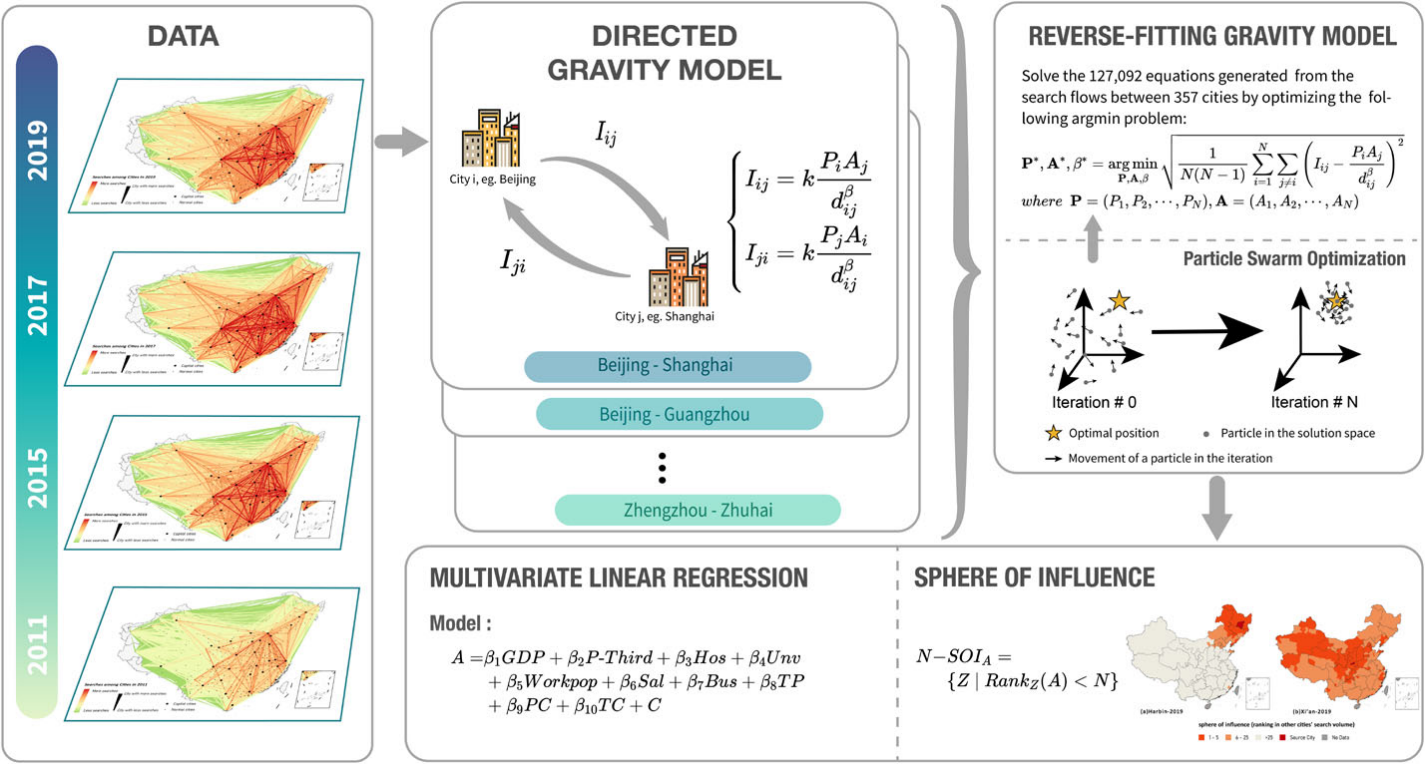
The proposed analytic framework

As aforementioned, the gravity model can be applied to estimate the theoretical sizes of the cities, known as gravitational attractions, given the interaction flows and distances between each city pair. To solve the problem of asymmetric flows, we introduce a directed modification of the gravity model, including two parameters, propulsion and attraction, for each city. Based on web search volumes and the distance between each pair of cities, we reversely fit the gravity model with a particle swarm optimization (PSO) approach. The derived values of attraction parameters stand for attractiveness of cities. Subsequent analyses are performed to explore the spatial pattern and temporal evolution of city attractiveness. Moreover, multivariate linear regression models are built to investigate the association between city attractiveness and socio-economic factors. Finally, the analysis of spheres of influence (SOI) provides another perspective to understand the change of major cities’ attractiveness. The proposed analytic framework is illustrated in Fig. 2.

### Directed gravity model

As an analogy to the law of universal gravitation, the gravity model was proposed for the modeling of spatial interactions. The simplified gravity model [24] can be expressed as

1 $$ G_{ij}=k\frac{S_{i} S_{j}}{d_{ij}^{\beta }}, $$

where $G_{ij}$ is the interaction intensity from city *i* to city *j*, $S_{i}$ and $S_{j}$ are sizes of the cities, $d_{ij}$ is the distance of certain measure between *i* and *j*, *β* is the distance decay coefficient, *k* is a constant.

Note that Eq. (1) is not suitable for modeling directed spatial interaction, where the interaction intensity $G_{ij}$ is not necessarily equal to $G_{ji}$. Most previous studies using reverse gravity model did not distinguish the two directions, where $G_{ij}$ denote sum of the flows in both directions. In our case, however, the search volume of two directions can be vastly different, so it is essential to distinguish the source and the target. Inspired by the idea of push-pull laws in migration studies [33] and the supply and demand formulation of gravity model in econometrics [34], we introduce two city parameters, propulsion and attraction, to replace the size parameters in the original model. We define the directed gravity model as follows,

2 $$ \textstyle\begin{cases} I_{ij}=k\frac{P_{i} A_{j}}{d_{ij}^{\beta }}, \\ I_{ji}=k\frac{P_{j} A_{i}}{d_{ij}^{\beta }}, \end{cases} $$

where $I_{ij}$ is the interaction flow from i to j, and $I_{ji}$ denotes the inverse flow. $P_{i}$ and $P_{j}$ are city propulsions, and $A_{i}$, $A_{j}$ are city attractions. The meaning of other parameters are the same as Eq. (1). This model assumes that the interaction intensity is proportional to propulsion of the source city and attraction of the target city. Note that the general gravity model [35], with different exponents for the source and target, is also capable of fitting directed interaction flows, yet its fitting performance on our dataset is significantly inferior (see Supplementary Discussion 3 in Additional file 1 for detailed comparison results).

The reversely fitting approach can be performed on the directed model as well, enabling us to use the search index data to estimate city propulsions and attractions. In specific, the model is fitted for search index data each year, where $I_{ij}$ in Eq. (2) denotes the annual average search index from city *i* to city *j*; $P_{i}$ and $A_{i}$ stand for the propulsion and attraction of city *i* in that year, respectively; $d_{ij}$ denotes the topological distance between city *i* and *j*. The topological distance of two cities is defined as their shortest path length on the adjacent graph. Two cities are considered adjacent if and only if they share a border as areal units. Hence, the distance between a city and its neighbor is 1, and between two non-adjacent cities with a common neighbor is 2 and so on. For cities on islands, we make Haikou linked to Zhanjiang (by Yuehai Railway) and Zhoushan to Ningbo (by Zhoushan Sea-crossing Bridge) so that the adjacent graph is connected. Topological distances are used instead of Euclidean distances for the following reasons. Regarding Euclidean distances, a point coordinate needs to be determined for each city area, which involves ambiguity [28]. Additionally, topological distances could mitigate the variation of city density across the country. Considering two cities with a distance of 500 km. In western China, they are probably neighbors, and people in one city could be very concerned with the other city. Yet in southeastern China, they could be separated by several cities, and people in one city might be unfamiliar with the other city at all. Therefore, we suppose topological distance is more consistent with cognitive distance of Internet users, which is more relevant in the case of web search than real distance. This notion is similar with rank-based friendship in social networks [36]. Moreover, a comparative experiment finds no significant difference in model fitness between the two distance measures (See Supplementary Discussion 4 in Additional file 1 for details).

### Reversely fitting the gravity model with PSO

Among existing methods for reversely fitting the gravity model, PSO is capable of handling large spatial interaction networks, and is applicable to the directed model. Therefore, we adopt PSO as our model solver. Particle swarm optimization (PSO) is a swarm intelligence approach to optimize non-linear continuous functions, proposed by Kennedy and Eberhart [37]. In PSO, the swarm is defined as a group of particles, each representing a position (i.e. a feasible solution vector) in the solution space. Initial positions of particles are generated randomly. In the iteration process, each particle records the best position it has ever searched as *Pbest*, while the swarm records the best position reached by all particles as *Gbest*. In a minimization context, for example, a position is better than another if lower value of the objective function is obtained. During each iteration, each particle flies to a new position, which is usually generated under the guidance of its *Pbest* and *Gbest* with randomness. After the movement, each *Pbest* and *Gbest* are updated. The algorithm exits when it reaches the maximum number of iterations, or the objective function falls below a predefined value. PSO is derivative-free, simple to implement, and intrinsically parallelizable. Similar to many other intelligent optimization methods, the theoretical foundation of PSO is relatively lack, yet its practical performance has been widely recognized [38].

This study uses PSO to minimize the root mean square error of the directed gravity model, as the objective function

3 $$ \text{RMSE}=\sqrt{\frac{1}{N(N-1)}\sum_{i\neq j} \biggl(I_{ij}- \frac{P_{i} A_{j}}{d_{ij}^{\beta }} \biggr)^{2}}, $$

where *N* is the number of cities, $I_{ij}$ stands for annual average search index from city *i* to city *j* $(1\le i,j\le N)$. The city parameters, $P_{i}$ and $A_{i}$, $i=1,2,\ldots ,N$, are obtained through PSO. We treat *β* as an input parameter and try different value at step 0.05. The *β* that produces the lowest RMSE is selected afterwards. Without loss of generality, let $P_{1}=A_{1}$ for the first city, so the solution vector should be $2N-1$ dimensional. In the case of 357 cities, the solution space has 713 dimensions.

The challenge of applying PSO to the optimization of a high-dimensional problem like this is nontrivial. In our experiment, the canonical PSO based on velocity update failed to find the optimal solution. To improve the algorithm performance, we incorporated features of two PSO variants, bare bones PSO with jumps (BBJ) and cooperative hybrid particle swarms (CPSO-H).

Based on bare bones particle swarms proposed by Kennedy [39], BBJ applies the following position update formula [40],

4 $$ x_{t+1}^{(d)}=\mathit{Gbest}^{(d)} + \alpha \epsilon \bigl\vert x_{t}^{(d)}-\mathit{Gbest}^{(d)} \bigr\vert ,\quad \epsilon \sim N(0,1), $$

where $x_{t}$ is the particle’s position after t iterations, $d=1,2,\ldots ,D$ is the component index of the *D*-dimensional solution vector, $N(0,1)$ stands for the standard normal distribution, *α* is a hyperparameter to control the exploration range. Besides, each dimension of each particle disobeys this formula at a probability $p_{J}$, which is called *jump*. When this happens, the new coordinate is generated from uniform distribution in $[X_{\min },X_{\max } ]$, where $X_{\min }$ and $X_{\max }$ are the lower and upper bound for each element of solution vectors, respectively. This feature allows the swarm to escape from local optima and avoid premature convergence. This research sets $\alpha =0.75$, and $p_{J}=0.001$, as the authors suggested.

Cooperative PSO [41] is proposed to deal with the curse of dimensionality, that the algorithm performance falls rapidly as dimensionality increases. In the CPSO-H algorithm, the solution space is divided into subspaces and a P-swarm is created to search in each subspace. At the same time, a Q-swarm is created to search in the whole solution space. The P-swarms and Q-swarm work alternatively, and cooperate through particle exchange. This mechanism allows Q-swarm to control the search direction globally, while P-swarms search subspaces thoroughly. Note that swarms in CPSO-H can take any updating method, including Canonical PSO and BBJ.

We implemented and tested four algorithms on average search index data in 2018, namely Canonical PSO, BBJ, CPSO-H + Canonical PSO and CPSO-H + BBJ. To reduce the computation time, we utilized GPU-based parallel computing with CUDA Toolkit 10.0. In our implementation, the objective function computations for each particle run in parallel on GPU cores, while the main thread does all the other work. Swarms with up to 4096 particles are used in our high-dimensional problem, which is greatly larger than the common setting of 20-50 particles [42]. Results show that CPSO-H + BBJ outperforms other algorithms, and is adopted in this study. (See Supplementary Discussion 2 in Additional file 1 for details on parameters and comparison of algorithms.)

### Multivariate linear regression

Given a city, the attractiveness reflects search behavior of individuals, including potential visitors and people interested in the city. It is related to many attributes and functions of the city, including economic development, traffic convenience, health care, educational services, employment, recreation and shopping services, ecological quality, administrative services, and housing [43]. As urban attractiveness in cyberspace is not limited by accessibility, traffic convenience is not a proper explanatory factor. Housing and environment are important to local residents, while Internet users in other cities have fewer concerns about these two attributes. Hence, six of nine city attributes are included in the model, including economics, recreation, health care, education, employment and administrative function. Additionally, the tourism function and business function could attract travelers and business visitors. They contribute to the web search volume of a city, and are essential to the explanation of city attractiveness. To sum up, eight categories of features are considered to explain the derived city attractiveness.

The influencing factors, the corresponding economic indicators, and their sources are given in Table 1. We use statistical indicators collected from *China City Statistical Yearbook 2019* [2], CEIC database [44], and the 7th national census [45]. Two of the selected factors, administrative service and tourism, are difficult to measure with statistical indicators due to the lack of data or inconsistency in statistic definitions among cities. Thus, we use two dummy variables to represent them based on official lists from the national government. The administrative variables are defined by the list of political cities, which includes 36 cities that are provincial capitals, municipalities directly under the central government, sub-provincial cities, or cities with independent planning status. These cities are more potential to receive beneficial policies from the central government, and get political attention from media. Cities with tourism function are defined with reference to the List of Top Tourist City of China by the National Tourism Administration of China [46], including 85 cities in our research. The two lists are provided in Supplementary Tables 4 and 5 in Additional file 1. In our model, we choose the normal cities as the base group and use three dummy variables, PC, TC, PT to represent political cities, tourism cities, and cities with both functions respectively.

**Table 1:** List of factors and corresponding variables

Factors	Indicators	Variables
Economics development	GDP in RMB yuan [2]	GDP
Recreation and shopping services	Proportion of tertiary sector [2]	P-Third
Health care	Total amount of beds in hospitals [2]	Hos
Educational services	Number of university students [44]	Unv
Employment	Working-age population [45]	Workpop
	Average salary [44]	Sal
Administrative services	Political city list	PC, PT
Tourism	Tourism city list [46]	TC, PT
Business development	Number of industrial enterprises [2]	Bus

A total of 254 cities are considered in the OLS regression, while other cities are excluded due to lack of data. Before the regression, the correlations between all statistical variables and city attractiveness are examined with Pearson correlation coefficient. Results show that all the variables are related at 0.01 significance (Supplementary Table 3 in Additional file 1). Meanwhile, all explanatory variables involved are rescaled with Z-score standardization. This step makes the regression coefficients comparable, so that the importance of different variables could be revealed. Finally, to ensure the completeness of the model, Ramsey RESET test [47] is performed to test variable omissions.

### Sphere of influence of a city

**Figure 3:**
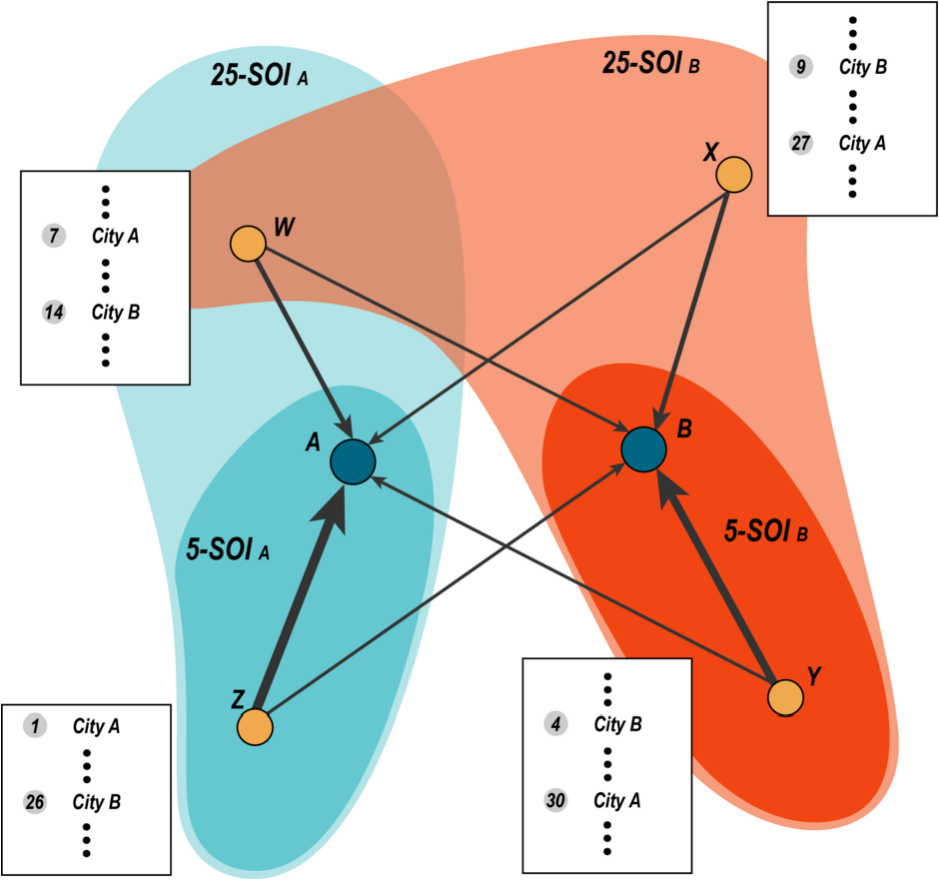
An Example of extracting city’s SOI. The figure shows the extraction of 5-SOI and 25-SOI of city *A* and city *B*, which are denoted by large blue nodes. The other four cities, *W*, *X*, *Y*, *Z*, are small yellow nodes, and only the search flows from such four cities to *A* and *B* are drawn. The list boxes beside the four nodes are the ranked lists of cities in which *W*, *X*, *Y*, and *Z* are interested. Note that for simplicity, searches from *A* and *B*, or to *W*, *X*, *Y*, *Z*, are not drawn

We can use the flow of web searches originated from other cities to a city *A* to delineate its sphere of influence (SOI). People in city *Z* may be interested in a number of cities, including *A*, which can be obtained based on search volumes from city *Z* to other cities. For example, if the search volume from *Z* to *A* is greater than those from *Z* to any other city, city *A* ranks first in the list of cities in which city *Z* is interested, and city *A* has the strongest influence on city *Z*. However, if *A*’s position is not so high, say 20, *A* has a relatively weak impact on *Z*. Hence, given a rank threshold *N*, the *N*-SOI of city *A* can be defined as

5 $$ N-SOI_{A}=\bigl\{ Z | \mathit{Rank}_{Z}(A)< N\bigr\} , $$

where *A*, *Z* are cities and $\mathit{Rank}_{Z}(A)$ means *A*’s rank in the list of cities in which *Z* is interested according to search volumes. Figure 3 illustrates the calculation of SOIs (5-SOI and 25-SOI) of two example cities *A* and *B*.

## Results

### Attractiveness of Chinese cities

By reversely fitting a directed gravity model for web search index data, we obtained the attractions and propulsions of 357 Chinese cities in 2019. The $R^{2}$ between real and predicted search index is 0.913, which indicates the data is well described by the directed gravity model. The estimated decay coefficient $\beta =0.4$. It is in accordance with previous results that interaction in cyberspace is still affected by distance decay [36], yet the intensity of decay is weaker than in real space [30]. Compared with propulsion that directly reflects the number of Internet users and their interests on other cities, attraction is found to be a more comprehensive indicator related to the population, function, and economic development of a city (Sect. 5.2). Hence, we focuses on city attractiveness hereafter.

**Figure 4:**
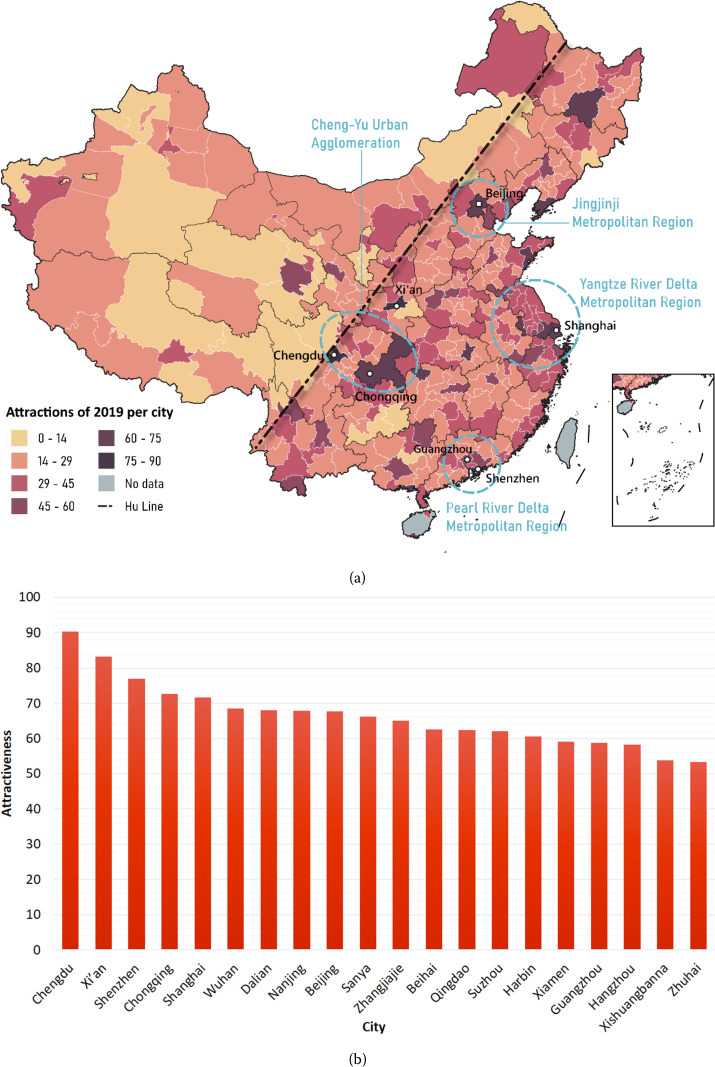
Cities’ attractiveness in 2019. (a) The Chinese map of cities’ attractiveness in 2019. (b) The top 20 attractive cities in 2019. The estimated city attractiveness is standardized so that the sum of all cities is 10000

The overall spatial distribution of the Chinese cities’ attractiveness (Fig. 4a) was highly east leant to the Hu Line [48]. Additionally, the four most famous metropolitan areas in China can be easily identified as the four clusters of cities with top attractiveness: (a) Jing-Jin-Ji Metropolitan Region in the north, including Beijing and Tianjin; (b) Yangtze River Delta in the east, including Shanghai and Hangzhou; (c) Pearl River Delta Metropolitan Region in the south, mainly covering the most densely urbanized and wealthiest regions in China; and (d) Western cities represented by Chengdu, Chongqing, and Xi’an, which developed fast recent years.

Note that Chengdu and Xi’an outperform traditional metropolises and become the most attractive cities (Fig. 4b). In addition to rapid economic development, high attractions of these cities may be attributed to rich tourism resources. In the top 20 list, several cities including Sanya (#10), Zhangjiajie (#11), Beihai (#12), and Xishuangbanna (#19), are relatively small cities, but gain much attentiveness due to their well-known city brands of appealing scenery or comfortable tourism services.

**Figure 5:**
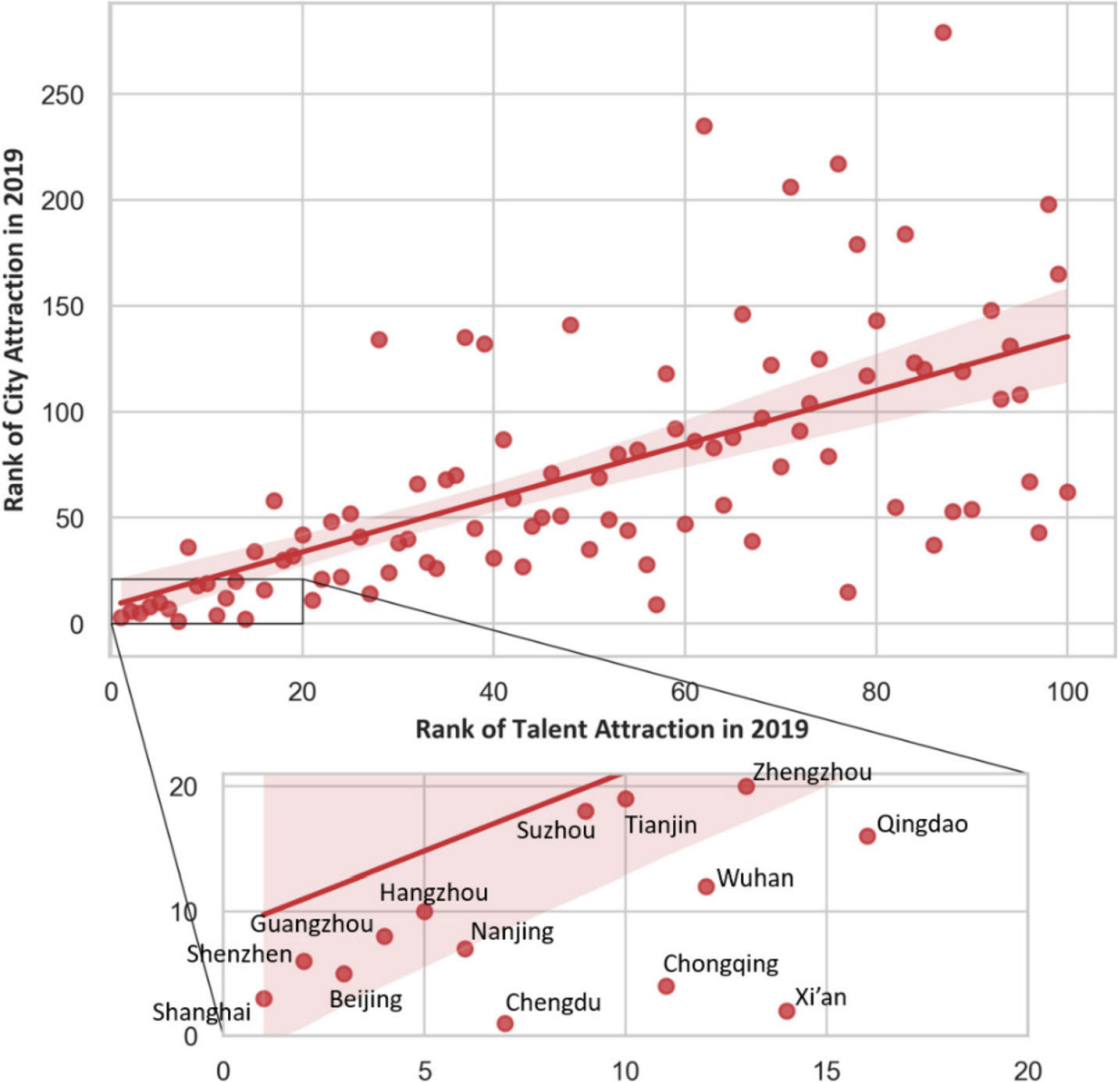
The correlation between city attractiveness and talent attraction. Cities in the top 20 in both rankings are labeled in the enlarged figure

The estimated attractiveness index can be cross validated by other city attractiveness indicators, such as the Top 100 cities for talent attraction in 2019 (published by [49], see Supplementary Table 6 in Additional file 1). The Spearman rank correlation coefficient between talent attraction and city attractiveness is 0.713 (Fig. 5, significant at 1% level). Note that 6 of top 20 cities ranked by estimated attractiveness are not in top 20 for talent attraction, such as Harbin and Sanya, which heavily depend on the tourism and hospitality industry. This implies that the estimated attractiveness takes the tourism factors into consideration and is a comprehensive proxy of the city’s real but immeasurable attractiveness.

### Driving factors of city attractiveness

Using the multivariate linear regression model, we evaluated the relationship between city attractiveness and city functions and attributes (Table 2). All city attributes and functions are positively related to the city attractiveness. The tertiary sector, health care, employment and tourism function are significant influencing factors. The result shows that the level of medical care has the greatest impact on the attractiveness of the city, followed by tourism. The uneven allocation of medical care resources [50] leads to the need to seek medical services across cities, which might increase the contribution of health care function to urban attractiveness. The high impact of tourism shows that people tend to target tourist destinations when searching the Internet. The average salary also has a positive impact on city attractiveness, showing that well-paid jobs can largely increase the attractiveness of the city. Gross domestic product (GDP) also contributes to the city attractiveness, indicating better economic development is more attractive to high-quality labor, businesses, and capital. The significance level of the Ramsey RESET test is 0.142, higher than 0.1. Therefore, we cannot reject the hypothesis that the model has no omitted variables, suggesting the completeness of our model.

**Table 2:** Regression results of city attractiveness

Variables	Coef.	Std Err.
GDP	0.100	0.095
P-Third	0.123^∗∗∗^	0.043
Hos	0.336^∗∗∗^	0.085
Unv	0.051	0.077
Workpop	0.113^∗∗^	0.046
Sal	0.151^∗∗∗^	0.053
TP	0.185^∗∗∗^	0.068
PC	0.042	0.037
TC	0.165^∗∗∗^	0.036
Bus	0.043	0.060
Constant	−0.002	0.034
Adjusted $R^{2}$	0.715	
F value	64.858	

Notes: ^∗∗∗^ and ^∗∗^ represent 1% and 5% significance levels, respectively.

### Changes of city attractiveness in the last decade

**Figure 6:**
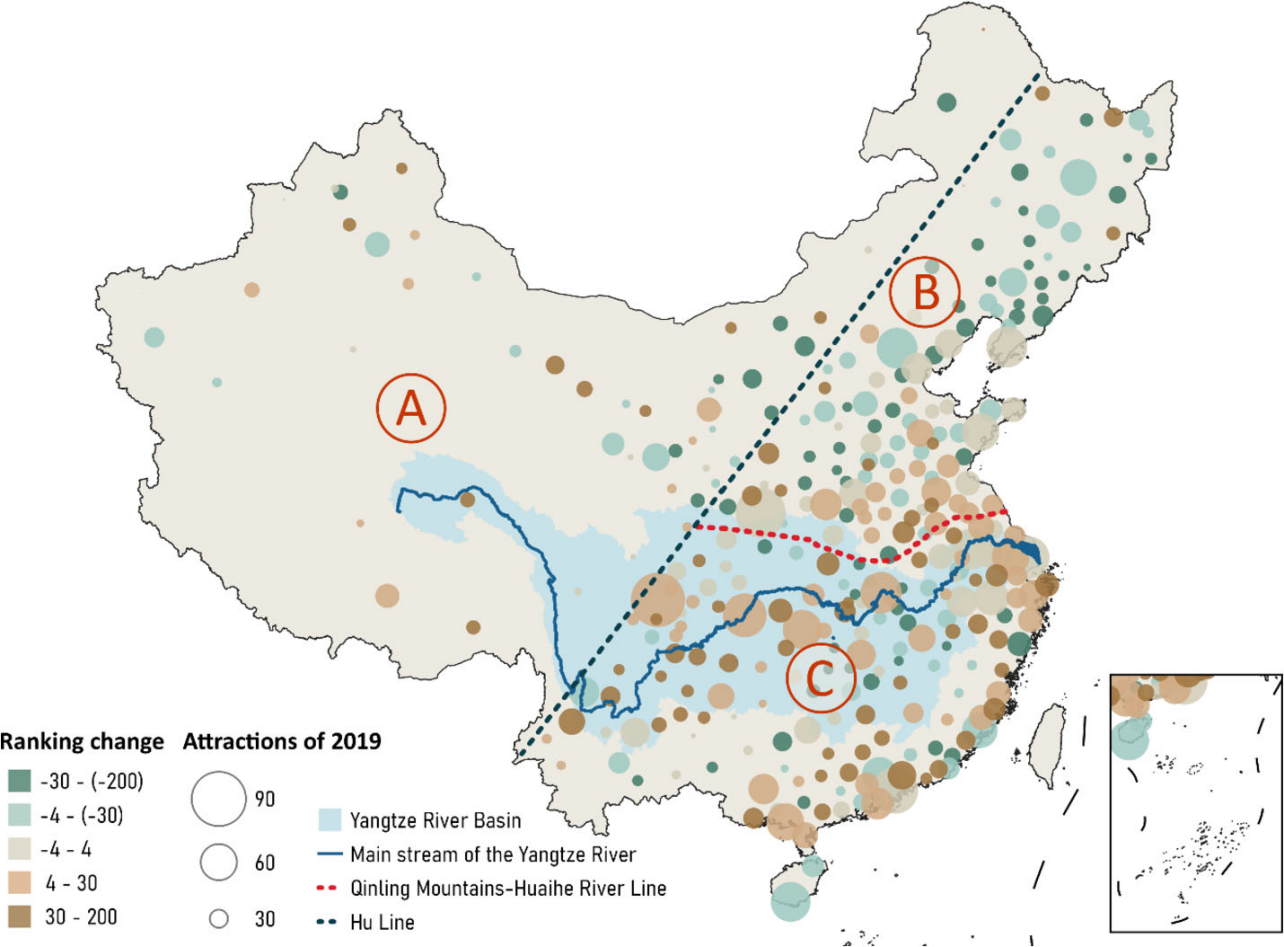
Changes of cities’ attractiveness from 2011 to 2019. Based on Hu Line and Qinling Mountains-Huaihe River Line, China is divided into three parts Western China (Zone A), Northern China (Zone B), Southern China (Zone C). In Zone A, 16.3% cities increase by more than 30 places in the ranking, while 16.3% cities decline by more than 30 places. However, in B and C, the two proportions are 6.3% vs. 23.9% and 20.0% vs. 9.8%, respectively. The obvious north-south contrast indicates a nationwide economic development trend

City attractiveness for each year from 2011 to 2018 is estimated with the same approach. The model fitness $R^{2}$ is all above 0.87, and the distance decay effect is in the range of 0.4 to 0.5 during the nine years (see Supplementary Discussion 2 in Additional file 1 for details). The temporal evolution of urban attractions from 2011 to 2019 revealed the features of the Chinese urbanization trend in the last decade (Fig. 6). The evolution of cities in northern China and southern China differ a lot, indicating that the northern cities’ development is slower than the southern cities. Northern China especially northeastern China is stuck in a quagmire of stagnation since China’s heavy industry economy is inevitably shrinking. In contrast, owing to fast development of light industries and high-tech industries, cities in southern China experienced a rapid growth in the past decade, widening the gap with the north part. Based on the two groups of cities, the traditional dividing line, i.e. the Qinling Mountains-Huaihe River Line, between the north and south parts, has gradually become the separatrix between two types of cities with opposite trends. Having made great advances, the cities along the Yangtze River, named Yangtze River Economic Belt, show the prospect to become China’s new growth pole.

### Major cities’ sphere of influence

**Figure 7:**
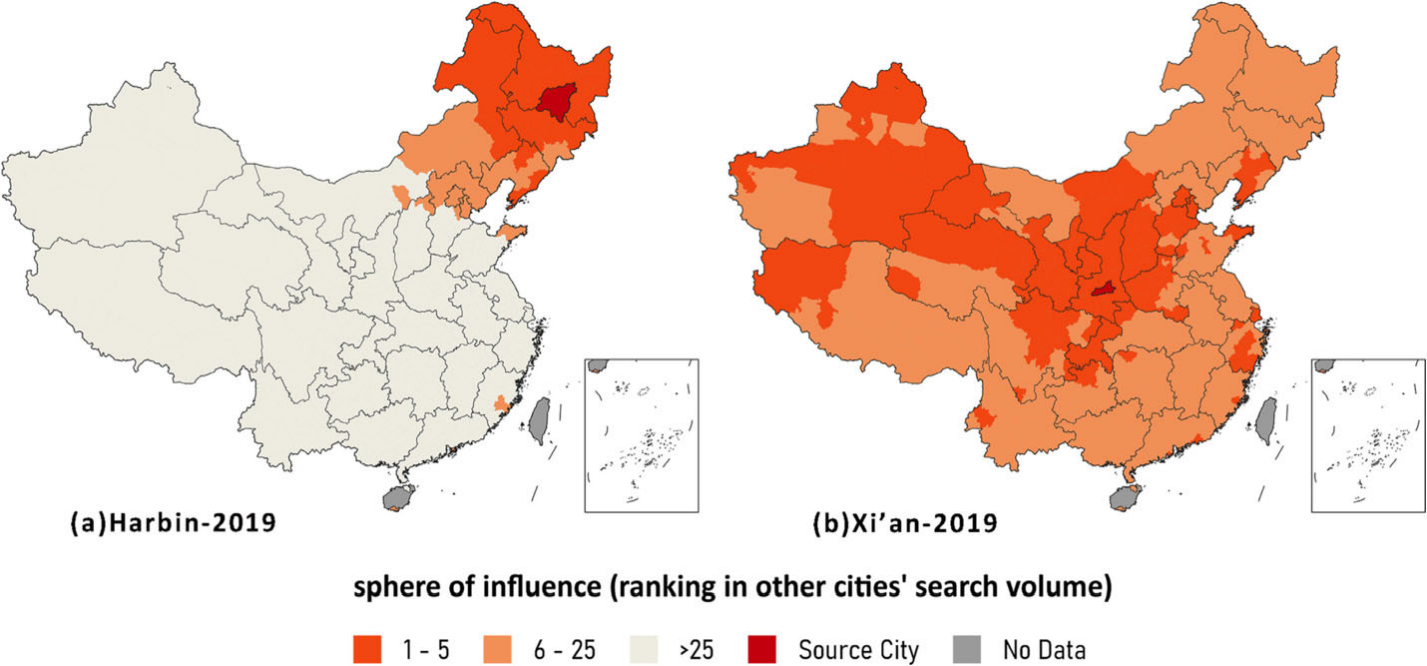
Harbin and Xi’an’s sphere of influence in 2019

**Figure 8:**
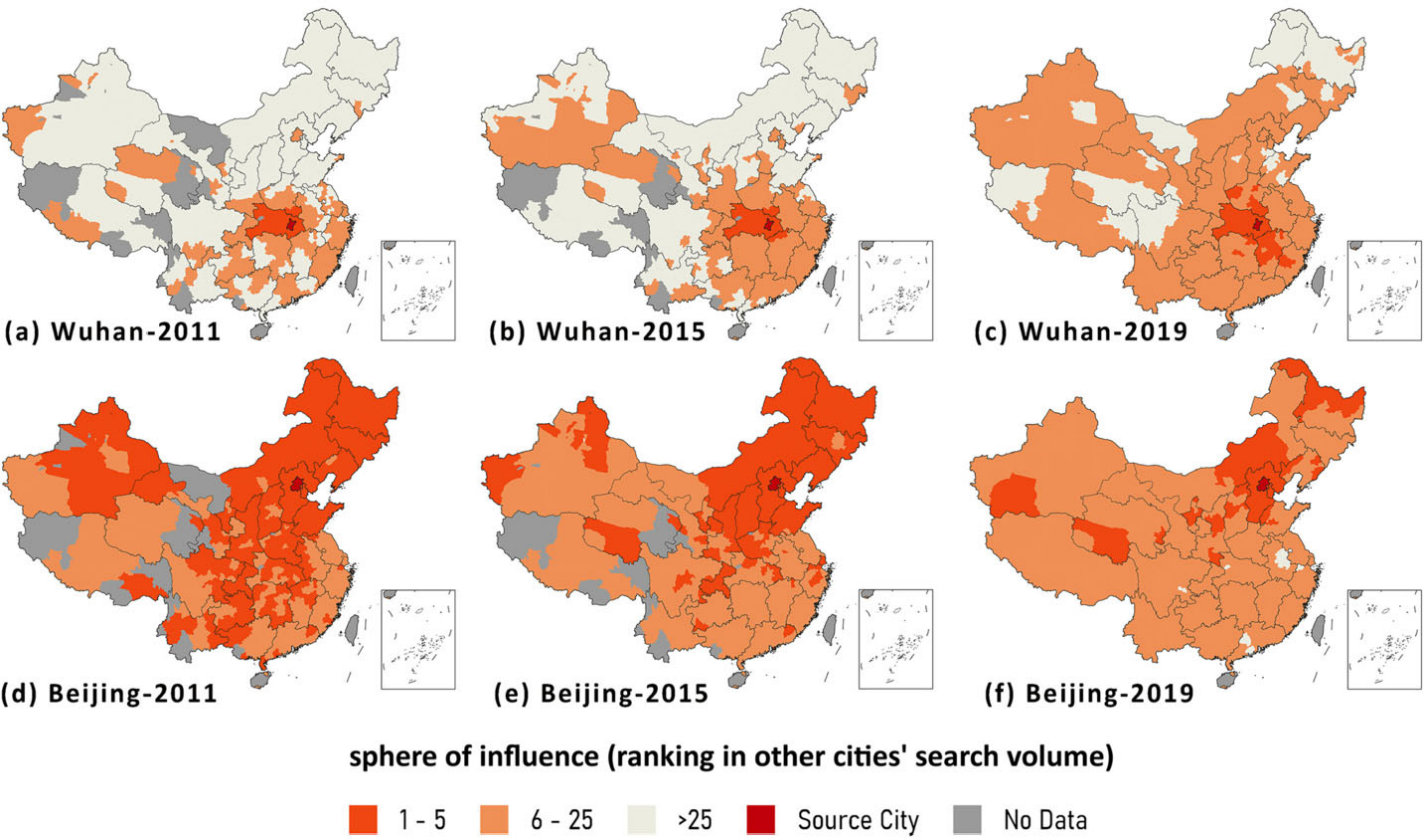
Beijing and Wuhan’s sphere of influence in 2011, 2015 and 2019

With the method described in Sect. 4.4, we can extract cities’ sphere of influence, named N-SOI. In this research, we assume that the 5-SOI delineates the sphere that is strongly influenced by the city and the 25-SOI depicts the region that was influenced by the city while the city’s impact is not so dominant. Figures 7 and 8 portray the 5-SOIs and 25-SOIs of several cities. In general, a big city has a nationwide SOI like Xi’an in Fig. 7b, while medium cities usually have local SOIs like Harbin in Fig. 7a. A greater SOI implies that the city attracts more attention and thus has a promising development prospect.

To identify cities with nationwide impact, we used a simple rule. If a city’s 25-SOI covers more than 60% of cities in a province, we say it covers that province; if a city’s 25-SOI covers more than 80% of provinces in China, then the city is viewed as a nationwide city. Following this criterion, we found 11 nationwide cities: Shanghai, Beijing, Nanjing, Zhangjiajie, Chengdu, Hangzhou, Wuhan, Shenzhen, Suzhou, Xi’an, and Chongqing. Among them, Zhangjiajie is famous for its tourism resources, and the other 10 cities are important tier-one cities.

Additionally, the change of SOI provides us an insight to foresee a city’s future. As shown in Fig. 8, the tendency of Wuhan and Beijing is quite different. In 2011, Beijing’s attractiveness control almost the whole China. However, when it comes to 2015 and 2019, its influence quickly declined (the area of orange zones) and step-by-step shrinks to merely northern China. Both the 5-SOI and 25-SOI of Wuhan only limited around Hubei Province, indicating a localized city in 2011. However, its 25-SOI gradually expands to whole China after 2011 and achieves national influence in 2019. Besides, in 2019, its 5-SOI also sprawls out of the Hubei Province, indicating that Wuhan is becoming a core city of the Yangtze River Economic Belt and has a promising future.

## Conclusions and discussion

An emerging proxy data source, search index data, is utilized to estimate the attractiveness of Chinese cities accounting for people’s interactions with cities in the cyberspace. This study takes advantage of its superiority on spatial coverage and temporal span. We obtain three main findings as follows. First, the web search indices are promising and reasonable data source with relatively high spatio-temporal resolution for evaluating urban systems. Second, given a city, its attractiveness and sphere of influence together quantify the potential to attract resources, in addition to attention, from other cities. Last, recognizing that high attractiveness and large sphere of influence imply positive future development, a notable trend is that most northern cities, including Beijing, are in decline.

The successful usage of particle swarm optimization algorithms on such high-dimensional problem further demonstrates its ability on solving the reverse gravity model, compared to previous studies [17, 26]. Our estimation and analysis of city attractiveness reveal the rise and fall of major cities and regions. These findings conform with the development of Chinese cities, and may indicate the development pattern of Chinese cities in the future. As web search data accumulate, the longitudinal search index would be a promising proxy for researchers and policy makers to understand and predict the future of China’s city system, as well as the economic development.

Our work might suffer the following limitations. First, ambiguity of a city’s name could induce bias on search index and estimated city attractiveness occasionally, as observed in our results. Besides, web users may search with a keyword related to the city rather than the city name. For example, someone interested in Beijing may search “Tian’anmen” (in Chinese). These searches are not counted in the search index, thus not reflected in our estimated city attractiveness. As most cities encounter the same problem, we assume that the impact of this issue is tolerable. Second, high search volume might come from negative public emergency rather than city attractiveness. By filtering out abnormal values, this effect could be largely mitigated. Third, the interests of those who seldom or even never surf the Internet is overlooked due to the sample bias of the data. As a result, the search index might reflect interests of young people more than the elderly, for example. Recent statistical data [51] show a rising trend on Internet penetration, and the proportion of elderly people in Internet users is growing as well. Therefore, it is hopeful that this issue might be mitigated gradually. We also suggest that the fusion of web search data and spatial interaction data in physical space, like human mobility data, might further resolve these limitations and produce more reliable results on the evolution of urban system.

Those limitations imply that a one-size-fits-all solution for measuring inter-city interactions as well as the derived city attractiveness might be impossible. Nonetheless, as the access to data set capturing the interactions between cities in both physical space and virtual space increases, it opens a possibility to synergize different interactions in the two spaces to fully understand city attractiveness. Furthermore, incorporating them is not straightforward, and the relationship between interactions needs to be considered. We may conjecture that the interplay between interactions in physical space and virtual space is either mutually complementary or exclusive. Moreover, there might exist spatially and/or temporally lagged effects between population movements in physical space and web search activities in virtual space. Empirical verification using spatio-temporal models will be a direction of further investigation. In a sense, our study introduces web search data as a promising (while being largely ignored) data source for the analysis of inter-city interactions, and appeals that more attentions should be paid to understanding city attractiveness in a coupled and multi-faceted fashion. As the big data are increasingly accessible, we see a lot of opportunities as well as challenges on this topic.

## Supplementary Information

Below is the link to the electronic supplementary material.

Including supplementary data, as well as detailed discussions on toponym ambiguity, comparison experiments on gravity model forms and distance metrics, implementation details and comparison experiments of particle swarm algorithms. (DOCX 49 kB)

## Data Availability

The Baidu Index dataset analyzed during the current study can be publicly retrieved from the official website. Preprocessed data and related source codes are available from the Github repository, https://github.com/s3pku/BaiduCityAttr.

## Abbreviations

BBJ: Bare bones particle swarms with jumps
CPSO-H: Cooperative hybrid particle swarm optimization
GDP: Gross domestic product
LP: Linear programming
PSO: Particle swarm optimization
RMSE: Root mean square error
SOI: Sphere of influence
