# Supplementary material for: Understanding China’s urban system evolution from web search index data
Source: EPJ Data Sci. 2022 Mar 28;11(1):20. doi: 10.1140/epjds/s13688-022-00332-y (PMC8959800; doi:10.1140/epjds/s13688-022-00332-y)
Supplement: Supplementary file 1 — Including supplementary data, as well as detailed discussions on toponym ambiguity, comparison experiments on gravity model forms and distance metrics, implementation details and comparison experiments of particle swarm algorithms. (DOCX 49 kB) [file 13688_2022_332_MOESM1_ESM.docx]

**Supplementary** **Data**

Table S1. Description Statistics of Annual Average Baidu Index between City Pairs

| **Year** | **max** | **mean** | **min** | **std** |
| --- | --- | --- | --- | --- |
| 2011 | 4050.422 | 25.642 | 0.156 | 48.269 |
| 2012 | 4164.016 | 25.178 | 0.156 | 48.291 |
| 2013 | 4018.534 | 29.906 | 0.156 | 56.874 |
| 2014 | 5098.732 | 39.321 | 0.156 | 76.874 |
| 2015 | 5517.066 | 43.011 | 0.156 | 82.016 |
| 2016 | 5136.132 | 43.986 | 0.156 | 78.071 |
| 2017 | 6340.521 | 47.025 | 0.156 | 87.279 |
| 2018 | 5236.51 | 47.821 | 0.156 | 80.620 |
| 2019 | 3195.203 | 45.608 | 0.156 | 74.456 |

Table S2. Internet Penetration Rate of Provinces in 2018

| **Province** | **Internet penetration rate (%)** | **Province** | **Internet penetration rate (%)** |
| --- | --- | --- | --- |
| Anhui | 50 | Jiangxi | 51 |
| Beijing | 75 | Jilin | 55 |
| Chongqing | 55 | Liaoning | 63 |
| Fujian | 69 | Ningxia | 52 |
| Gansu | 46 | Qinghai | 54 |
| Guangdong | 74 | Shaanxi | 55 |
| Guangxi | 50 | Shandong | 57 |
| Guizhou | 49 | Shanghai | 74 |
| Hainan | 51 | Shanxi | 57 |
| Hebei | 56 | Sichuan | 48 |
| Heilongjiang | 52 | Tianjin | 65 |
| Henan | 48 | Tibet | 47 |
| Hubei | 57 | Xinjiang | 55 |
| Hunan | 50 | Yunnan | 43 |
| Inner Mongolia | 54 | Zhejiang | 67 |
| Jiangsu | 58 |  |  |
| Source: Annual Report on China Internet Development (2018), ChinaNetCenter | | | |

Table S3. Correlation coefficients between indicators and city attractiveness

| **Indicator** | **Pearson’s Correlation** |
| --- | --- |
| GDP | 0.749^***^ |
| Number of industrial enterprises | 0.534^***^ |
| Average salary | 0.549^***^ |
| Working population | 0.479^***^ |
| Number of university students | 0.714^***^ |
| Proportion of tertiary sector | 0.560^***^ |
| Total amount of beds in hospitals | 0.730^***^ |
| Note: ***, **, *respectively represent 1%, 5%, 10% significance levels. | |

Table S4. List of Cities with Political Function

| **Cities with Political Function** | | | |
| --- | --- | --- | --- |
| Beijing | Hohhot | Xiamen | Xining |
| Chengdu | Jinan | Shanghai | Yinchuan |
| Dalian | Kunming | Shenzhen | Changchun |
| Fuzhou | Lhasa | Shenyang | Changsha |
| Guangzhou | Lanzhou | Shijiazhuang | Zhengzhou |
| Guiyang | Nanchang | Taiyuan | Chongqing |
| Harbin | Nanjing | Tianjin |  |
| Haikou | Nanning | Urumqi |  |
| Hangzhou | Ningbo | Wuhan |  |
| Hefei | Qingdao | Xi’an |  |

Table S5. List of Cities with Tourism Function

| **Cities with Tourism Function** | | | | | | | |
| --- | --- | --- | --- | --- | --- | --- | --- |
| Anqing | Chongqing | Haikou | Jinhua | Ningbo | Shijiazhuang | Wuxi | Yulin |
| Anshan | Dalian | Hangzhou | Kaifeng | Puyang | Shiyan | Xiamen | Zhangjiajie |
| Baoji | Danton | Harbin | Kunming | Qingdao | Suzhou | Xi'an | Zhangzhou |
| Baotou | Daqing | Hefei | Leshan | Qinhuangdao | Tai'an | Xianyang | Zhaoqing |
| Beihai | Datong | Huangshan | Lhasa | Quanzhou | Taiyuan | Xuzhou | Zhengzhou |
| Beijing | Foshan | Huizhou | Liuzhou | Sanya | Tianjin | Yangzhou | Zhenjiang |
| Benxi | Fushun | Jiangmen | Ma'anshan | Shanghai | Turpan | Yantai | Zhongshan |
| Changchun | Fuzhou | Jiayuguan | Mudanjiang | Shantou | urumchi | Yichang | Zhuhai |
| Changsha | Guangzhou | Jilin | Nanchang | Shaoxing | Weihai | Yichun |  |
| Chengde | Guilin | Jinan | Nanjing | Shenyang | Wuhan | Yinchuan |  |
| Chengdu | Guiyang | Jingzhou | Nanning | Shenzhen | Wuhu | Yueyang |  |

Table S6. Top 100 cities for talent attraction in 2019 by Zhaopin.com

| **City** | **Rank** | **Talent Attraction** | **City** | **Rank** | **Talent Attraction** |
| --- | --- | --- | --- | --- | --- |
| Shanghai | 1 | 100 | Huzhou | 51 | 6.9 |
| Shenzhen | 2 | 85.3 | Weihai | 52 | 6.9 |
| Beijing | 3 | 78.7 | Shaoxing | 53 | 6.7 |
| Guangzhou | 4 | 75.1 | Urumqi | 54 | 6.4 |
| Hangzhou | 5 | 69.5 | Zhenjiang | 55 | 6.3 |
| Nanjing | 6 | 53.2 | Luoyang | 56 | 6.3 |
| Chengdu | 7 | 46.9 | Sanya | 57 | 6.1 |
| Jinan | 8 | 39.4 | Tangshan | 58 | 6 |
| Suzhou | 9 | 37.3 | Zhangjiakou | 59 | 5.4 |
| Tianjin | 10 | 35.9 | Quanzhou | 60 | 5.2 |
| Chongqing | 11 | 33.4 | Wuhu | 61 | 5.2 |
| Wuhan | 12 | 32.9 | Pingxiang | 62 | 5.2 |
| Zhengzhou | 13 | 31.6 | Kaifeng | 63 | 5.2 |
| Xi'an | 14 | 29.9 | Taizhou | 64 | 5.1 |
| Dongguan | 15 | 29.6 | Xianyang | 65 | 5 |
| Qingdao | 16 | 28.5 | Zibo | 66 | 4.9 |
| Foshan | 17 | 25.3 | Qinhuangdao | 67 | 4.8 |
| Changsha | 18 | 24.8 | Mianyang | 68 | 4.7 |
| Wuxi | 19 | 24.2 | Zhuzhou | 69 | 4.7 |
| Hefei | 20 | 22.1 | Salt City | 70 | 4.6 |
| Xiamen | 21 | 20.8 | Weinan | 71 | 4.5 |
| ningbo | 22 | 19.5 | Suqian | 72 | 4.4 |
| Shijiazhuang | 23 | 18.9 | Taizhou | 73 | 4.3 |
| Zhuhai | 24 | 16 | Cangzhou | 74 | 4.3 |
| Fuzhou | 25 | 15.2 | Huai'an | 75 | 4.3 |
| Changzhou | 26 | 14.2 | Meishan | 76 | 4.2 |
| Dalian | 27 | 13.9 | Harbin | 77 | 4.2 |
| Langfang | 28 | 13.7 | Water | 78 | 4.2 |
| Kunming | 29 | 13.5 | Xuchang | 79 | 4.1 |
| Huizhou | 30 | 12.6 | Dezhou | 80 | 4.1 |
| Nanchang | 31 | 12.6 | Zunyi | 81 | 4.1 |
| Taiyuan | 32 | 11.3 | Lhasa | 82 | 4 |
| Guiyang | 33 | 11.2 | Xingtai | 83 | 3.9 |
| Shenyang | 34 | 10.9 | Jining | 84 | 3.9 |
| Jiaxing | 35 | 10.6 | Jiangmen | 85 | 3.8 |
| Nantong | 36 | 10.3 | Xining | 86 | 3.8 |
| Zhongshan | 37 | 10.1 | Jinzhong | 87 | 3.7 |
| Wenzhou | 38 | 10 | Lianyungang | 88 | 3.7 |
| Baoding | 39 | 9.9 | Luzhou | 89 | 3.7 |
| Xuzhou | 40 | 9.6 | Yinchuan | 90 | 3.6 |
| Weifang | 41 | 9.3 | Heze | 91 | 3.5 |
| Yantai | 42 | 9.1 | Qingyuan | 92 | 3.5 |
| Nanning | 43 | 8.7 | Zhaoqing | 93 | 3.5 |
| Yangzhou | 44 | 8.1 | Rizhao | 94 | 3.4 |
| Changchun | 45 | 7.9 | Yibin | 95 | 3.4 |
| Hohhot | 46 | 7.9 | Handan | 96 | 3.4 |
| Haikou | 47 | 7.4 | Zhanjiang | 97 | 3.3 |
| Jinhua | 48 | 7.1 | Zhoukou | 98 | 3.3 |
| Linyi | 49 | 7.1 | Shangrao | 99 | 3.3 |
| Lanzhou | 50 | 6.9 | Yichang | 100 | 3.3 |

**Supplementary Discussions**

1. **Toponym Ambiguity**

Similar to other studies that involve toponyms, our study faces the issue of toponym ambiguity. When one searches with a city name as a keyword, it may not refer to the city itself. Liu et al. [28] discussed two cases of toponym ambiguity. In some cases, it refers to something that has semantic relationships with the city. For example, the city name “Huangshan” may refer to Huangshan Mountain, the famous scenic spot in the city of Huangshan. As tourism attractions is a part of the city attractiveness, cases like this are acceptable. Nevertheless, it may overestimate the city attractiveness compared to cities with names different from their famous scenic spots (Taishan Mountain in Tai’an City, for example). In other cases, a city name may appear as keyword without semantic relationships with the city. Ali is a prefecture-level region in Tibet Autonomous Region, while the name “Ali” is sometimes viewed as the abbreviation of Alibaba, a well-known Chinese hi-tech company, or even Muhammad Ali, the famous boxer. Another example is Hainan Autonomous Prefecture of Qinghai Province, whose name is the same with Hainan Province. In both cases, cities with ambiguous names often have abnormally high search index values, which is influenced by toponym ambiguity obviously. However, a systematical detection and elimination of such cases is not straightforward. We did not modify our data for this issue, but will consider its influence when explaining our results.

1. **Details and Comparison of PSO Algorithms**

We implemented and tested four algorithms with annually-averaged search index data in 2018, namely Canonical PSO, BBJ, CPSO-H + Canonical PSO and CPSO-H + BBJ. For Canonical PSO, we follow the suggested parameter selection that $c_{1}=c_{2}=2$, and $w$ decreases linearly from 0.9 to 0.4 during the iterations. The boundary reflects particles for a certain distance ($\Delta d=1)$. That is to say, if a particle’s coordinate on certain dimension falls below $X_{min}$, set it to $X_{min}+\Delta d$; if it exceeds $X_{max}$, set it to $X_{max}-\Delta d$. This feature is added to handle the condition that particles stagnate at the boundary after hitting it. For BBJ, we use $\alpha=0.75$, $p_{J}=0.001$, and a reflecting boundary is not necessary. For CPSO-H, as a solution vector is 713-dimensional, we chose $K=23$, so that all sub-vectors are 31-dimensional. Each swarm has 1024 particles. For CPSO-H algorithms, there are 24 swarms and a total of 24576 particles. CPSO-H algorithms run for 3000 iterations, and non-CPSO-H algorithms run for 10000 iterations. All algorithms use $X_{min}=0.1$, $X_{max}=100$, while initial positions of particles are generated in $[5,70]$. For Canonical PSO algorithms, we limit particle velocity by $V_{max}=10$. The distance decay coefficient $\beta=0.4$. Each algorithm is repeated for 10 times.

The minimum, mean and standard error of RMSE are listed in Table S8. BBJ outperforms Canonical PSO for its stronger ability to escape from local optima. CPSO-H improves the result combined with either Canonical PSO or BBJ, which exhibits better stability as well. BBJ+CPSO-H appears to be the best solver for our large-scale reverse gravity model, which is adopted in our study.

Table S7. RMSE results of four PSO algorithms

| Algorithm | Minimal  RMSE | Mean  RMSE | Std.Error of  RMSE |
| --- | --- | --- | --- |
| Canonical PSO | 20.2620 | 21.2641 | 1.4124 |
| BBJ | 19.6746 | 19.7698 | 0.1011 |
| Canonical PSO  +CPSO-H | 19.6732 | 19.6832 | 0.0082 |
| BBJ+CPSO-H | 19.6696 | 19.6698 | 0.0001 |

When estimating the model parameters with CPSO-H+BBJ for each year from 2011 to 2019, we use swarms of 4096 particles and 10000 iterations to acquire more precise results. Other configurations are the same as stated above, except that the scale factor $k$ in directed gravity model is introduced as the solution would be too small if $k=1$. The boundary $X_{min}$ and $X_{max}$ are adjusted accordingly. The optimal $\beta$ varies from 0.4 to 0.5 during the seven years, and the $R^{2}$ values are no less than 0.87 (Table S9).

Table S8. Summary of model estimations for 2011-2019

| Year | 2011 | 2012 | 2013 | 2014 | 2015 | 2016 | 2017 | 2018 | 2019 |
| --- | --- | --- | --- | --- | --- | --- | --- | --- | --- |
| Cities | 322 | 322 | 322 | 322 | 322 | 322 | 357 | 357 | 357 |
| $\boldsymbol{\beta}$ | 0.45 | 0.40 | 0.45 | 0.50 | 0.45 | 0.40 | 0.40 | 0.40 | 0.40 |
| RMSE | 10.744 | 10.398 | 15.406 | 21.809 | 21.747 | 20.674 | 20.166 | 19.670 | 18.143 |
| $\mathbf{R}^{\boldsymbol{2}}$ | 0.870 | 0.886 | 0.874 | 0.871 | 0.889 | 0.898 | 0.910 | 0.913 | 0.914 |

1. **Comparison with the General Gravity Model**

Another possible candidate to fit directed interaction flow is the general gravity model

$G_{ij}=k\frac{P_{i}^{a}P_{j}^{b}}{d_{ij}^{\beta}},G_{ji}=k\frac{P_{j}^{a}P_{i}^{b}}{d_{ij}^{\beta}}$ (S1)

where $a, b$ are global constants. Like the simplified model, this model has one parameter $P_{i}$ for each city, yet with different exponents for the source and the destination. Actually, our proposed model is a relaxation of model (S1), where $a, b$ may be different for each city. With more parameters, our model would fit any data at least as well as model (S1).

Results show that our model performs significantly better than model (S1). We used the search index data in 2018 with 357 cities to compare the two models. CPSO-H + BBJ algorithms is used to solve the two models with the same settings (4096 particles, 10000 iterations). Model (S1) reaches an RMSE of 26.441 with $R^{2}=0.842$, while our model gets an RMSE of 19.670 with $R^{2}=0.913$. The results indicate that our model successfully depicts the pattern of city name search. At the same time, the separation of city propulsion and attraction in spatial interaction enables us to analysis city attraction more precisely, which lays the basis of this study.

1. **Comparison of Topological and Euclidean Distances**

The distance decay item $f(d_{ij})=d_{ij}^{-\beta}$ in the gravity model depends on the choice of distance measures. The standard option is Euclidean distance, which is straightforward for nodes represented as geographical points. For interactions between regions, however, this approach is ambiguous on the choice of representative points. Additionally, the density of cities in Western China is much lower than Eastern China. With topological distances, the variation of city density across the country is mitigated.

We compared the two distance measures by optimizing the reverse gravity model in 2018. The adopted model optimizer, CPSO-H+BBJ, is used for both cases. To be precise, the Euclidean distance is spherical distance on the earth surface. The geographical coordinates of cities are collected through a geocoding API provided by amap.com. The city coordinates are located in downtown area of the city, usually at the city government. The spherical distance between two cities is calculated by

$D=Rarccos(cos\varphi_{1}\cos\varphi_{2}\cos\left( \theta_{1}-\theta_{2} \right)+\sin\varphi_{1}\sin\varphi_{2})$ (S2)

where *R* is the average radius of the earth, $(\theta_{i}, \varphi_{i})$ is the longitude and latitude of city $i$, $i=1,2$.

With regard to model fitness, the model using Euclidean distance reaches an RMSE of 19.040, with $R^{2}=0.919$，which is slightly better than model using topological distance (RMSE = 19.670, $R^{2}=0.913$). The difference of fitting performance is not significant, thus is not considered as evidence for superiority of Euclidean distance. As for the estimated city attractiveness, results from two distance metrics coincides well, with a Pearson’s correlation coefficient of 0.986 and a Spearman's rank correlation coefficient of 0.977, both significant at 0.01 level. Eight out of the Top 10 attractive cities given by the two distance metrics coincide (Table S10), with minor changes in ranks. These results demonstrated the stability of our method. Generally, we observe slight increase in the attractiveness of western cities (like Urumqi) and some northeastern cities (like Harbin), and slight decrease in southeastern cities, after switching to Euclidean distances. This is consistent with the fact that western cities become “closer” in topological distance and southeastern cities more distant.

Table S9. Top 10 attractive cities in topological and Euclidean distance

| Topological distance Top 10 | Standardized  Attractiveness | Euclidean distance Top 10 | Standardized  Attractiveness |
| --- | --- | --- | --- |
| Chengdu | 116.76 | **Chengdu** | 120.28 |
| Xi’an | 91.99 | **Xi’an** | 94.27 |
| Shanghai | 91.14 | **Chongqing** | 94.10 |
| Chongqing | 85.79 | **Shanghai** | 85.77 |
| Beijing | 85.67 | **Beijing** | 83.61 |
| Shenzhen | 82.35 | **Shenzhen** | 73.28 |
| Nanjing | 75.50 | Harbin | 72.44 |
| Guangzhou | 72.20 | **Nanjing** | 70.54 |
| Sanya | 71.41 | Zhangjiajie | 66.81 |
| Hangzhou | 68.82 | **Guangzhou** | 66.35 |
